# Technique for central aortic cannulation in extensive aortic dissection

**DOI:** 10.1308/003588412X13373405386015c

**Published:** 2012-09

**Authors:** TK Rajab, JD Schmitto, R Gallegos

**Affiliations:** ^1^Brigham and Women’s Hospital, Boston, MA,US; ^2^Hannover Medical School,Germany

## BACKGROUND

Cardiopulmonary bypass for aortic dissection repair is most frequently established via the peripheral arteries. Central cannulation in the context of aortic dissection was first described in 1998.^1^ In this situation, the difficulty lies in determining whether the true lumen or the false lumen is being cannulated. Previous authors have described cannulation of the proximal aorta^2–6^ and confirmation of the wire in the true lumen by ultrasonography in a non-dissected segment of the distal aorta.^6^ However, this is not possible in complete dissection of the aorta down to the iliac bifurcation.

## TECHNIQUE

In this situation, access to the descending aorta can be gained via the Seldinger technique and the wire carefully passed retrograde towards the aortic valve. Cannulation of the true lumen can be confirmed by transoesophageal echocardiography with the wire floating freely by the aortic valve. Once the position of the wire in the true lumen is confirmed, a cannula can be placed over the wire.

## DISCUSSION

Extensive dissection of the aorta with concurrent contraindications for peripheral cannulation poses a particular problem for cannulation since it can be difficult to ensure cannulation of the true lumen. In these cases, the technique described above with proximal passing of the wire and confirmation of its position in the true lumen by transoesophageal ultrasonography can be a useful method.
